# Special Issue: “Ceramics and Construction Materials”

**DOI:** 10.3390/ma14154204

**Published:** 2021-07-28

**Authors:** Teresa Mangialardi

**Affiliations:** Department of Chemical Materials Environment Engineering, Faculty of Civil and Industrial Engineering, Sapienza University of Roma, Via Eudossiana 18, 00184 Roma, Italy; teresa.mangialardi@uniroma1.it

The variety of material classes engaged for constructions is very wide, ranging from naturally occurring substances, such as stone materials and wood, to manufactured products such as inorganic binders, ceramic bricks, adhesives, metals, composites like concrete, bituminous or fibre-reinforced materials. Often, construction materials and ceramics are considered traditional materials with well-established compositions and manufacturing processes. Supports of this belief are the use of clay minerals as main raw materials and the presence of high-temperature treatments in the industrial manufacturing processes of bricks, tiles, hydraulic binders, pipes and refractories.

In the recent years, all the above types of materials have been requested for continuous enhancement of their functional properties and performances. Furthermore, the increasing environment concerns and demand for developing sustainable and circular economy have promoted significant changes in the selection of alternative raw materials and in the development of new or improved production processes. Primary goals of the innovation have been the preservation of natural resources, the reduction of pollutant emissions and, at the same time, tailoring of the products characteristics. Much has been done but the potential for much more to be studied and developed about all the above materials is still equally wide. 

At his launching, the aim of the Special Issue “Ceramics and Construction Materials” was to collect contributions from the research community illustrating the current direction and the recent advances of the search in the field of the above materials, with a special look to the new green and ecofriendly materials.

The papers published in this Special Issue deal with different aspects of manufacturing, characterization and maintenance of ceramics and construction materials. They give a screenshot of the present status of the world research in the field. Contributions coming from several countries, like Australia, China, Czech Republic, Korea, Latvia, Poland, Portugal, Slovakia and UK, well illustrate how ceramics and construction materials are becoming more and more different from traditional ones and how their final properties largely depend on engineering optimization. 

Several published articles are focused on binders and concrete [1,2,3,4,5,6,7], so showing the great scientific interest associated to these materials. Attention has been reserved to the low-carbon cements, i.e., cementing binders produced with low greenhouse emissions [1,2]. Among these binders are fly ash belite cements and alkali activated materials, the latter representing a potential instrument for the future sustainable development of the construction industry. In study [1], it has been demonstrated that addition of Na_2_O, as an alkali activator, to fly ash accelerates the formation of poorly crystallized hydrates in the hydrothermal synthesis of the cement. This, in turn, allows the formation of a good pozzolanic material even with the lower temperatures of the process. In addition, the application of a kinetic model based on the Jander’s equation reveals to be appropriate for predicting the evolution of the hydrothermal process. Very interesting results were obtained by Gonçalves et al. [2], which focused their research on the production and characterization of one-part alkali activated material obtained exclusively from industrial byproducts and wastes. At this purpose, granulated blast-furnace slag and exhausted sands from biomass boilers were used as components of mortars. Besides the significant compressive strength of the materials developed, the interest of the proposed application is in avoiding industrial wastes landfilling, so reducing the related environmental impact. 

As far as the properties of cementitious mixes obtained by means of innovative technologies, in article [3] the effect of a particular kind of mixing water, i.e., the hydrogen nanobubbles water (HNBW) has been investigated on Portland cement mortars. The authors studied the effect of HNBW concentration on compressive and flexural strengths of mortars and showed its beneficial effects. The improvement in the mechanical properties of mortars is related both to the size reduction of bubbles with increasing their concentration in water and the increased probability of cement particles to hydrate when impacting bubbles with reduced sizes. Papers [4,5,6,7] deal with reinforced or not-reinforced concrete mixes and investigate different aspects of their performances. A finite element model is used in [4] to simulate the dynamic responses of a concrete-filled tubular steel member under the combined action of axial compression, creep and axial impact. A calculation formula for the peak impact load under axial compression considering creep is proposed, which can help in the design of the impact resistance of this kind of structure. Paper [5] reports about the effects of various types of recycled microfiller, such as brick, concrete and glass powders, on concrete properties. It is well-known that the use of inert powders offers both economic and ecological benefits to the concrete industry. Moreover, using powders having potential pozzolanic effects may have positive extra effects on the properties of concrete. After a testing time of 3 years, the ecofriendly concretes investigated, containing a high dosage of filler, have been judged suitable for use both in terms of improved durability and mechanical properties. Papers [6,7] are focused on the mechanical properties of fibre-reinforced concrete. Glass and polypropylene fibres have been used by Ahmed and Jia [6] as reinforcing materials. They have demonstrated that hybrid fibres could aid in enhancing the durability of concrete by reducing the permeability at low fibre volume. Steel or polypropylene fibres have been investigated by Sadowska-Buraczewska et al. [7] as reinforcing materials of a concrete layered structure with fibres in the compressed layer of bending slabs. The benefit of using polypropylene with respect to steel fibres has been demonstrated in terms of increased load capacity and stiffness.

A work related to metallic construction materials has been developed by Li et al. [8]. In this study, concerning copper-bearing steel, the authors have shown that a high-temperature thermal treatment (1150 °C) is able to promote the copper segregation onto the steel surface. A uniform copper layer is formed without discontinuity with respect to the bulk matrix. The studied treatment is proposed not only to prepare copper-clad steel, but also for in situ-formation of composite materials by interface bonding.

Papers [9,10] are focused on the production of ceramics with enhanced performance characteristics while paper [11] deals with the maintenance of ceramic porous bricks. The first two studies explore the possibility of using waste materials as modifiers of the traditional formulas of specific manufactured, with the purpose of reducing the firing temperature and, consequently, the production costs too. In the first work, Shishkin et al. [9] have added green glass waste to illitic clay to produce ceramics having lower porosity and improved resistance to failure. Further advantages of using such a waste for the production of ceramics are related not only to the appreciable economic benefits coming from lowering the firing temperature, but also to the environmental benefits deriving from the reuse of municipal wastes. A process utilizing industrial byproducts has been studied in work [10] to produce high-alumina aggregates with the perspective to be used as a new mullite grog. Considering that the quality of grog determines to a large extent the properties of the final product, the optimization of aggregates is of a great importance for the production of specific and modern refractories. A formula has been developed by Zemánek and coworkers [9] featuring similar properties as the available commercial products, but with reduced production expenses. The last paper dealing with ceramics [11] has a strong practical relevance since its findings, coming from a laboratory study on appositely clogged porous bricks, may be applied to daily maintenance of permeable pavements exposed to rainwater runoff and could positively affect their service life cycle. 

In conclusion, the published papers demonstrate the scientific and technological relevance of the topics dealt with. As evidence of the readers interest, most of them have registered at the time of this Special Issue publication more than 700 readings. Furthermore, the published articles have reached on the whole 36 literature citations.
